# Reference miRNAs for colorectal cancer: analysis and verification of current data

**DOI:** 10.1038/s41598-017-08784-3

**Published:** 2017-08-21

**Authors:** E. Danese, A. M Minicozzi, M. Benati, E. Paviati, G. Lima-Oliveira, M. Gusella, F. Pasini, G. L Salvagno, M. Montagnana, G. Lippi

**Affiliations:** 1 Clinical Biochemistry section, Department of Neurosciences, Biomedicine and Movement Sciences, University of Verona, Verona, Italy; 2 Colorectal & Peritoneal Oncology Centre, The Christie NHS Foundation Trust, Manchester, Manchester, M20 4BX United Kingdom; 3 Laboratory of Pharmacology and Molecular Biology, Oncology Department, Rovigo General Hospital, 45027 Trecenta, Italy; 4 Department of Medical Oncology, Rovigo Hospital, 45100 Rovigo, Italy

## Abstract

MicroRNAs (miRNAs) hold great promise in cancer research. The use of appropriate reference miRNAs for normalization of qPCR data is crucial for accurate expression analysis. We present here analysis and verification of current data, proposing a workflow strategy for identification of reference miRNAs in colorectal cancer (CRC). We performed a systematic review of studies aimed to identify stable reference miRNAs in CRC through high-throughput screening. Among the candidate miRNAs selected from the literature we excluded those predicted to target oncogenes or tumor suppressor gene. We then assessed the expression levels of the remaining candidates in exosomes, plasma and tissue samples from CRC patients and healthy controls. The expression stability was evaluated by box-plot, ∆Cq analysis, NormFinder and BestKeeper statistical algorithms. The effects of normalisers on the relative quantification of the oncogenic miR-1290 was also assessed. Our results consistently showed that different combinations of miR-520d, miR-1228 and miR-345 provided the most stably expressed reference miRNAs in the three biological matrices. We identified suitable reference miRNAs for future miRNA expression studies in exosomes plasma and tissues CRC samples. We also provided a novel conceptual framework that overcome the need of performing *ex novo* identification of suitable reference genes in single experimental systems.

## Introduction

MicroRNAs (miRNAs) are short non-coding RNA fragments involved in post-transcriptional regulation of gene expression. In physiological conditions, miRNAs regulate a kaleidoscope of biological pathways including cell differentiation, proliferation and survival. Changes in miRNA expression profiles are frequently associated with abnormal cellular functions. Different expression patterns have hence been observed in a variety of diseases, including cancer. MiRNAs play an important role in the multistep processes of carcinogenesis, by targeting either oncogenes or tumor suppressor genes. The current research based on miRNA expression has been broadened into many types of tumors. Among different malignancies, miRNA expression in colorectal cancer (CRC) has been deeply analyzed for establishing its potential role as diagnostic, prognostic and predictive tool^1–5^. Although several technologies have been used for enabling high-throughput and sensitive miRNA profiling, reverse transcription quantitative real-time polymerase chain reaction (RT-qPCR) is still regarded as the method of choice due to higher sensibility and specificity. In qPCR experiments, data are usually reported as relative expression quantification, and the signal of the target miRNA in a treatment group is conventionally compared to that of an untreated control population. More specifically, the PCR-derived cycle threshold (Cq) of a target miRNA is compared with that of a stably expressed endogenous miRNA obtained from the same sample. The difference between these values is referred to as the ∆Cq value. The process of normalization is required to reduce the potential bias attributable to sample-to-sample non-biological variations introduced throughout the total testing process, from sample preparation to amplification and analysis. Therefore, the ultimate purpose is to accurately distinguishing between biological changes and experimentally induced variation. The possibility to report data as ∆Cq value also enables a more reliable comparison of data obtained within the same experiment and even among different experiments.

Due to the fact that an universal reference gene to be suitably used for all types of tissue does not exist so far, the normalization procedure has represented one of the most important and challenging issues in qPCR experiments. A huge number of miRNA expression studies has been published, but many data have been recently questioned since the endogenous reference genes which are commonly used for normalization may be unstably and heterogeneously expressed^6^. It has also been emphasized that the use of an inaccurate procedure for normalization may be one of the most relevant causes of limited overlap between findings from similar studies in the same pathological condition. The most accurate approach recently endorsed entails a selection of stably expressed reference to be used in each specific experiment, according to the tissue of interest and by means of high-throughput technologies^7, 8^. Nevertheless, this approach is often difficult to carry out due to the substantial cost of microarray assays.

Many efforts in miRNA research are currently focused on developing a standardized procedure for data normalization of miRNA expression, based on reference gene(s) selection. Due to the lack of universal consensus, some specific experimental workflows have been proposed^9–12^. Notably, Schwarzenbach and coauthors recently highlighted that research studies based on qPCR miRNA quantification for identifying candidate reference genes, should be anticipated by an extensive literature review of previous studies bas transcription polymerase chain ed on similar patient populations, on the analysis of the same biological matrix and using an identical process of sample management^13^. The suitability of using the identified miRNAs for a specific type of samples should then be validated in a sample subset.

Despite a large number of miRNA expression studies has been published in CRC patients, a critical review of reference genes that can be reliably used to normalizing miRNA expression is lacking to the best of our knowledge. Therefore, the aim of this study was to identify the most suitable candidate miRNAs among those already investigated, and then verifying their stability in tissue, plasma and exosomes of both controls and CRC patients.

## Results

### Literature review

**Figure 1:**
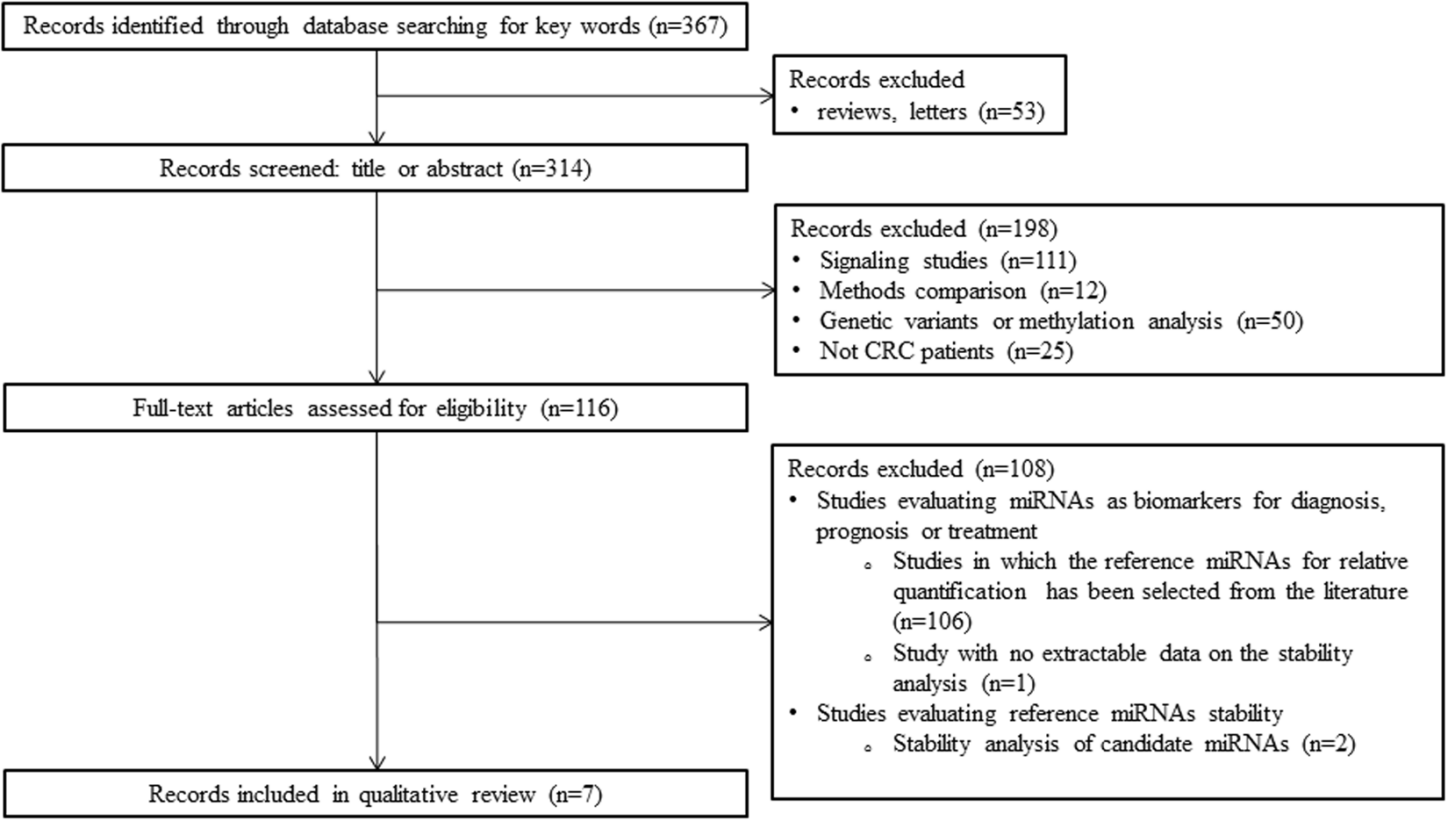
Flow diagram of the study selection process.

We carried out an electronic search for identifying studies which assessed the stability of reference miRNAs in CRC. The initial electronic search allowed to identify a total number of 367 items (Figure 1). Overall, 51 review articles and three letters were immediately excluded. Then, 198 articles were also excluded after reading titles or abstracts. In particular, 17 of these studies analyzed the effect of dietary or environment on miRNAs, 94 were signaling studies, 12 were method comparison studies, 25 did not included CRC patients, 50 studies evaluated the association between CRC and functional variants of miRNAs or promoter methylation of target miRNAs. Full text was analyzed for study eligibility from the remaining 116 publications. In 109 of these studies the purpose was to establish the potential role of miRNAs in CRC diagnosis, prognosis and treatment. When reported, target miRNAs have been quantified by using housekeeping miRNA selected from the literature (mostly miR-16, RNU6B, RNU48, cel-39, 18SRNA). In some cases the most stably reference miRNA was selected among a list of candidates^14, 15^. In one study miR-451 was chosen as reference gene from high throughput microarray data, but the selection method was not reported^16^. Nine studies were designed to identify reference genes for qPCR analysis in CRC patients. Two of these performed the stability study by screening a list of candidate miRNAs selected from the literature (U6, miR-16, miR-24, miR-142-3p, miR-19b and miR-192and miR-16, RUN6B and miR191) and ought to be excluded^15, 17^. The remaining 7 studies met our predefined inclusion criteria and were finally included in our qualitative review^7, 8, 18–22^. The most important details of these studies are shown in Table 1, along with the list of the candidate reference miRNAs ranked as the most stable by the different research groups.

**Table 1:** Characteristics of retrieved studies aimed to identify reference miRNA for CRC experiments through high-throughput screening and subsequent qPCR validation.

**First Author**	**Country**	Sample type	Discovery phase				Validation phase				Method used to assess miRNAs stability	Most stably expressed reference miRNAs
			Measurement method	cases and control samples	miR selection criteria	Candidate reference genes	Measurement method	cases and control samples	miR selection criteria	Candidate reference miRNAs		
Zheng G *et al*. 2013^18^	China	serum	Miseq sequencing (Illumina)**-740 miRNAs**	Three pooling serum samples: **-**colorectal adenocarcinoma (n = 30) **-**colorectal adenoma (n = 25) **-**healthy controls (n = 30)	miRNAs (a) having at least 50 copies in the three pooling samples (b) showing no differential expression among three groups (P > 0.05)	miR-103b miR-484 miR-16-5p miR-3615 miR-18a-3p miR-197-3p miR-191-5p miR-151a-3p miR-26a-5p miR-4446-3p miR-221-3p miR-93-5p miR-3184-3 RNU6	RT-qPCR Instrument: ABI PRISM 7500 Sequence Detection System using the SYBR PrimeScript Output: average values of triplicate Cq	Not matched samples (n = 125): **-**colorectal adenocarcinoma (n = 45) **-**colorectal adenoma (n = 40) **-**healthy controls (n = 40)	(a) the miRNAs must be expressed in all samples (b) mean Cq of the miRNAs must be below 35 (c) no evidence for differential expression among the three groups (P > 0.005)	miR-103b miR-484 miR-16-5p miR-3615 miR-18a-3p miR-191-5p RNU6	GeNorm and NormFinder	-miR-191-5p -RNU6
Chang KH *et al*. 2010^8^	Ireland	tissue	TaqMan Human MicroRNA array card(Applied Biosystems/Life Technologies) **-384 miRNAs**	Matched samples (n = 20): **-**tumour tissues from patients with stage II CRC (n = 10) **-**normal tissues from patients with stage II CRC (n = 10)	miRNAs whose expression pattern was similar to the global mean expression	let-7a miR-26a miR-345 miR-425 miR-454 + RNU48 Z30 miR-16	RT-qPCR Instrument: 7900HT Fast Real- Time PCR System (Applied Biosystems) Output: average values of triplicate Cq	Not matched samples (n = 74): **-**35 tumour specimens **-**39 normal tissues	MiRNAs with equivalent expression of reference genes between tumour and normal tissues (fold change cutoff of logarithmised Cq = 3)	let-7a miR-16 miR-26a miR-345 miR-425 miR-454 RNU48 Z30	GeNorm and NormFinder	-miR-345 -miR-16
Boisen MK *et al*.2015^19^	Denmark	tissue	TaqMan Array Human MicroRNA A + B Cards Set version 3.0 (Applied Biosystems/Life Technologies) - **754 miRNAs**	Not matched samples (n = 240): **-**mCRC (n = 203) -healthy controls (n = 37)	20 miRNAs whose expression pattern correlated best with global mean expression	miR-103a-3p miR-152-3p miR-132-3p miR-27a-3p miR-140-5p miR-30b-5p miR-339-5p miR-331-3p miR-374a-5p miR-652-3p miR-335-5p miR-185-5p miR-30c-5p* miR-151-5p miR-106b-5p miR-199a-3p miR-28-5p* miR-425-5p miR-26a-5p miR-24-3p	RT-qPCR Instrument: 7900HT Fast Real-Time PCR System. (Applied Biosystems) Output: Cq values	Not matched samples (n = 240): **-**mCRC (n = 203) **-**healthy controls (n = 37)	MiRNAs with Cq values ≤ 32 and with a 260/230 nm absorbance ratio ≥ 1.5. MiRNAs with less than 5% undetermined measurements * miR-30c-5p and miR-28-5p excluded because they were in the same miRNAs family as other miRNAs with better stability scores	miR-103a-3p miR-152-3p miR-132-3p miR-27a-3p miR-140-5p miR-30b-5p miR-339-5p miR-331-3p miR-374a-5p miR-652-3p miR-335-5p miR-185-5p miR-151-5p miR-106b-5p miR-199a-3p miR-425-5p miR-26a-5p miR-24-3p	GeNorm MiRNAs having a stability value ≥ 0.15	miR-103a-3p miR-152-3p miR-132-3p miR-27a-3p
Hu J *et al*. 2014^20^	China	plasma	Human microRNA microarrays (Agilent Technologies) - **723 miRNAs**	80 control subjects: **-**healthy controls (n = 34) -**-**CHB patients (n = 21) **-**cirrhosis patients (n = 25) 171 cancer patients: **-**HCC (n = 57) **-**CRC (n = 41) **-**lung cancer (n = 73)	MiRNAs with (a) Cq values ≤ 35 in at least 80% of samples and (b) stable value < 0.5 (by Genorm, Normfinder and CV) (c) miRNas controls commonly used in literature	miR-1225-3p miR-1228 miR-30d miR-939 miR-940 miR-188-5p miR-134 + miR-16 miR-223 let-7a RNU6B	RT-qPCR Taqman MicroRNA Assays (Applied Biosystems) Output: average values of triplicate Cq	92 control subjects: -healthy controls (n = 30) -CHB patients (n = 31) -cirrhosis patients (n = 31) 92 cancer patients: -HCC (n = 31) -CRC (n = 31) -lung cancer (n = 30)	MiRNAs with (a) Cq values ≤ 35 in at least 80% of samples (b) specific Taqman probe for RT-qPCR	miR-1225-3p miR-1228 miR-30d miR-939 miR-188-5p miR-134 miR-16 miR-223	GeNorm and NormFinder miRNAs with (a) the minimum CV value and stability values (b) no statistical difference between early and late tumor stages	miR-1228
Rice J *et al*. 2015^21^	Kentucky (USA)	plasma	Microfluidic array technology (Applied Biosystems) **-380 miRNAs**	CRC patients (not characterized)	miRNAs expressed in > 50% of samples and miRNAs selected from the literature	Let-7a Let-7d Let-7g RNU48 RNU6 miR-520d-5p miR-16 miR-191 miR-223 miR-484	RT-qPCR Step-One Plus RT-PCR System (Life Technologies, Carlsbad, CA)	-CRC (n = 20) -adenoma (n = 11) -breast cancer (n = 10) -lung cancer (n = 10) -pancreatic cancer (n = 10) healthy controls (n = 12)	miRNAs with uniform expression in all samples	RNU6 miR-520d-5p miR-16 miR-191 miR-223 miR-484	miRNAs with the greatest expression and the narrowest SD	-RNU6 -miR-520d-5p
Peltier HJ *et al*. 2008^22^	Texas (USA)	tissue	mirVana miRNA Bioarrays V1 (Ambion) -**287 miRNAs** RT-qPCR	“horizontal scan”: -13 individual normal flash-frozen human tissue RNAs -5 flash-frozen human tumor (including CRC)/NAT RNA pairs	(a) the miR must be highly expressed in most, if not all, of the samples (b) the miR must be consistently expressed, as measured by the modified z-score (c) only one representative from a given miR family should be considered (d) the miR must be a target of a commercially available qRT-PCR assay at the time of the work	miR-191 miR-93 miR-106a miR-17–5p miR-25 miR-16 let-7a miR-24 miR-103 miR-99a miR-23a miR-107 + RNU6 5s rRNA	mirVana miRNA Bioarrays V1 (Ambion) -**287 miRNAs** RT-qPCR	“vertical scan”: -12 flash-frozen human lung tumor/NAT RNA pairs -16 FFPE human lung tumor/NAT RNA pairs	miRNAs with uniform expression in all sample groups	miR-191 miR-24 miR-103 miR-17–5p let-7a mir-106 miR-16	GeNorm and NormFinder	NAT group: miR-191 (or miR-191 and miR-25) Tumour group: miR-103 Two sample groups combined: miR-191 and miR-25
Eriksen AHM *et al*. 2016^7^	Denmark	tissue	Microfluidic array technology- OpenArray Human MicroRNA Panel (Life Technologies) **-750 miRNAs**	-rectal cancer tissue (n = 10) -stromal tissue (n = 10)	(a) relative quantification (RQ) close to 1:1 (where the reference biological group is stromal tissue) (b) miR detected in more than 70% of all replicates of all samples (c) Cq values between 18 and 28 (d) no intentions of including the miR as a target in our future projects	let-7g miR-193a-5p miR-27a miR-645 + RNU6	RT-qPCR Custom TaqMan1Array MicroRNA Cards (Life Technologies) Expression Suite data-analysis	2 validation studies: -rectal cancer tissue (n = 25) and adjacent stromal tissue (n = 25) (A) -rectal cancer tissue (n = 28) and normal rectal mucosa (n = 28) (B)	miRNAs with uniform expression in all samples	let-7g miR-193a-5p miR-27a + RNU6	NormFinder miRNA with high expression stability	-let-7g miR-193a-5p miR-27a

CHB, chronic hepatitis B; CV, coefficient of variability; FFPE, fixed paraffin-embedded; HCC, hepatocellular cancer; mCRC, metastatic colorectal cancer; NAT, normal adjacent tissue; NPC, nasopharyngeal; RT-qPCR, real-time quantitative reverse-transcription polymerase chain reaction; SD, standard deviation

### MiRNAs selection

Because miRNAs function through post-transcriptional gene repression, we retrieved from miRTarBase^23^ the experimentally validated target genes of candidate reference miRNAs selected from the literature: hsa-let-7g, hsa-miR-103a, has-miR-1228, hsa-miR-16, hsa-miR-132, hsa-miR-152, has-miR-191, hsa-miR-193, hsa-miR-25, hsa-miR-27a, hsa-miR-345 and hsa-miR-520d. Only miRNA-target interactions validated by western blot analysis or Luciferase report assay were used for the in silico analysis. Weak functional miRNA-target interactions, identified by high-throughput sequencing technologies, were excluded. Altogether, these 12 miRNAs cumulatively targeted 178 genes, 21 of which were targeted by multiple miRNAs (ranging from 2 to 4), thus enhancing the total number of miRNA:mRNA interactions to 202 (Additional file 1). To gain a specific insight in cancer context, we parsed the target genes of candidate miRNAs and compared them against the list of ‘tumour suppressor genes’ or ‘oncogenes’, respectively (retrieved from the UniProt Knowledgebase^24^. See Methods section for details).

Seven out of 12 miRNAs target at least two oncogene or tumor suppressor gene. More specifically, miR-16 showed the largest number of onco/tumor suppressor genes (n = 13) followed by miR-27a (n = 8), let-7g (n = 6), miR-25 (n = 6), miR-152 (n = 4), miR-132 (n = 3) and miR-103a (n = 2). Oncogenes with multiple miRNA interactions were *BCL1* (miR-16, miR-27a and miR-152), *KRAS* (let-7g, miR-16, miR-27a and miR-152), *BMI-1* (let-7g, miR-16), *MYB* (miR-103a, miR-16). Tumor suppressor genes with multiple miRNA interactions were: *PTEN* (miR-103a, miR-25), *RECK* and *TP53* (mir-16, miR-25). The remaining 5 candidate miRNAs (miR-191, miR-193a, miR-345, miR-520d and miR-1228), showing no interactions with any known oncogene or tumor suppressor gene, were selected for the experimental study. The spike in control cell-39 was also included for subsequent analyses due to its broad use in literature as external reference control.

### Expression levels of candidate reference miRNAs

The six candidate reference miRNAs displayed a wide expression range, with Cq values comprised between 15.0 and 33.9 in the entire study sample. The expression levels of each miRNA in the three different matrices is shown in Table 2. Except miR-345, the expression values of which were relatively low in exosomes and moderately abundant in both plasma and tissue samples, all the other miRNAs displayed median Cq values sufficiently homogenous between the three matrices. The most highly expressed miRNA was miR-191, which exhibited a median Cq values of 22.4 in exosome, 22.9 in plasma and 21.9 in tissue samples, respectively. In contrast, other miRNAs were found to be less abundant; for example miR-520d displayed a median Cq values of 30.3, 30.1 and 29.7 in exosome, plasma and tissue samples, respectively, whereas miR-345 displayed a median Cq value of 30.5 in exosomes.

**Table 2:** Expression levels of candidate reference miRNAs in different matrixes from CRC patients and healthy controls.

	Exosome		Plasma		Tissue	
miRNAs	Median (range)	∆Cq	Median (range)	∆Cq	Median (range)	∆Cq
cell-39	25.6 (19.8–28.3)	8.5	24.3 (20.2–29.0)	8.8	23.8 (21.4–29.0)	7.6
mir-345	30.5 (26.4–33.0)	6.6	25.5 (21.8–30.2)	8.4	24.6 (22.9–27.4)	5.3
mir-1228	28.2 (26–30.7)	4.7	27.9 (24.9–29.8)	4.9	25.3 (21.9–29.5)	7.6
mir-191	22.4 (16.3–33.9)	17.6	22.9 (16.1–31.9)	15.9	21.9 (18.1–27.4)	9.4
mir-193	25.5 (19.1–33.4)	14.3	21.3 (17.2–25.2)	8	23.0 (19.9–25.3)	5.4
mir-520d	30.3 (28.1–33.7)	5.6	30.1 (27.9–32.4)	4.5	29.7 (26.2–31.7)	5.5

**Figure 2:**
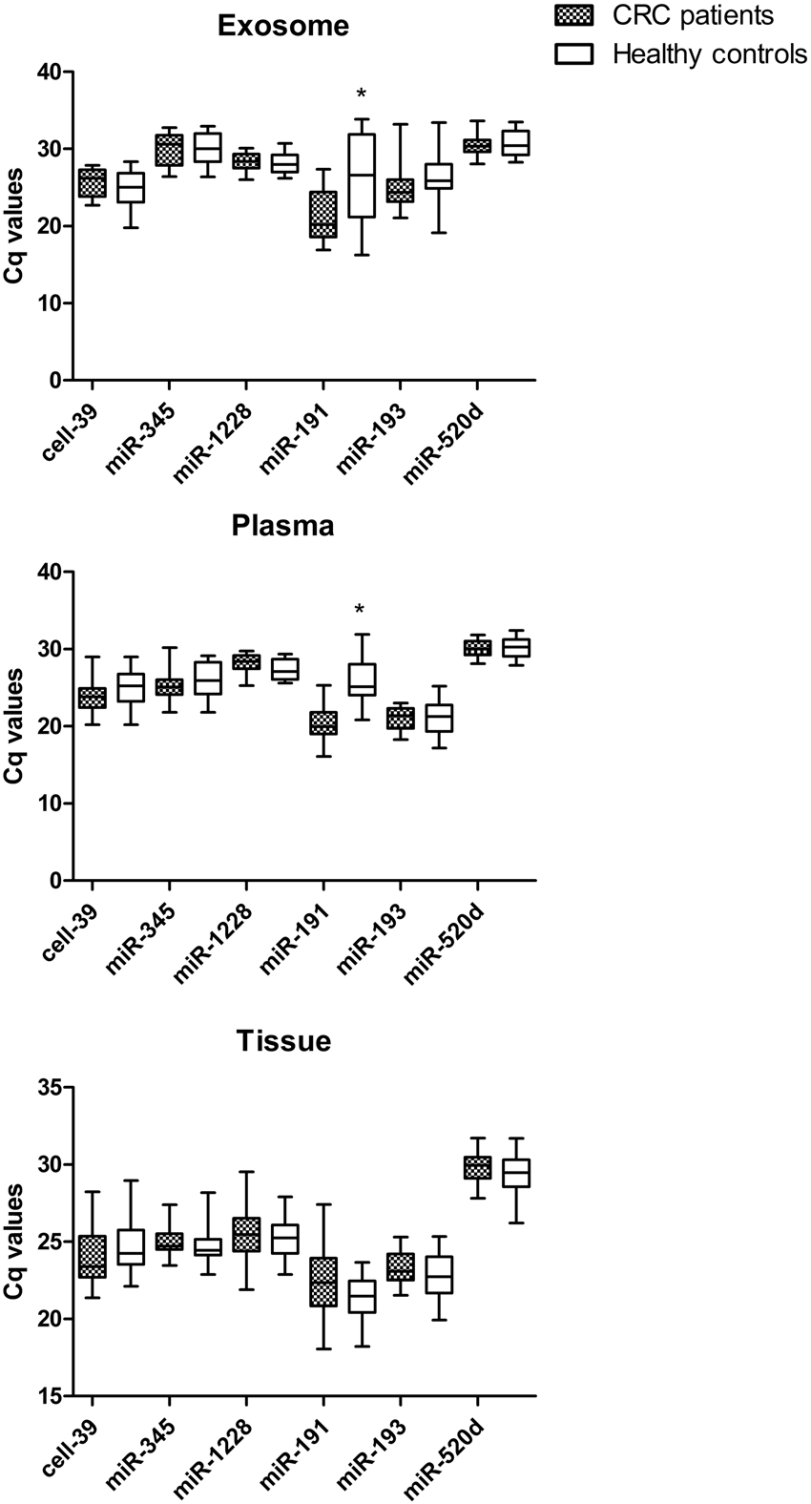
Expression levels of candidate reference miRNAs. Box and whisker plot displaying the Cq values for each putative reference miRNA. There is no significant difference in the expression level of cell-39, miR-345, miR-1228, miR-193 and miR-520d, between tumour and normal samples in exosomes, plasma and tissue samples. miR-191 showed a significant difference between exosome and plasma samples from CRC and healthy controls (p = 0.015 and﻿ p < 0,0001).

The range of Cq values for miR-191 was rather broad in both exosome and plasma samples, with ∆Cq values above 15 cycles. High ∆Cq were also found for miR-193 in exosomes (14.3 cycles). All the other miRNAs displayed narrow Cq range in all sample sets. According to the ∆Cq values, the most stably expressed miRNAs were miR-1228 in exosomes, miR-520d in plasma samples and miR-193 in tissues (Table 2). As shown in Fig. 2, the box - plot analysis does not reveal significant differences of expression levels of all but one candidate reference miRNAs (Figure 2). As predicted from analysis of ∆Cq, the expression level of miR-191 was higher in exosome and plasma samples from CRC patients than in those of healthy controls (p = 0.015 and p < 0.0001 respectively). Due to this different expression level, miR-191 was excluded from further stability analysis in exosome and plasma samples.

### Expression stability of candidate reference miRNAs

The variable stability of the selected reference genes was evaluated using NormFinder and BestKeeper algorithms. The rankings of reference genes by analysis of stability value generated by the two algorithms are summarized in Tables 3 and 4. Lower stability values characterize greater stability. The summarized data indicates that the NormFinder and BestKeeper algorithms produce similar rank orders for candidate reference miRNAs expression stability.

**Table 3:** Ranking of candidate reference miRNAs using NormFinder algorithm.

Rank	Exosome		Plasma		Tissue	
	Gene name	Stability value	Gene name	Stability value	Gene name	Stability value
1	miR-520d	0,260	miR-520d	0,227	miR-520d	0,191
2	mir-1228	0,277	mir-193	0,352	mir-1228	0,239
3	miR-345	0,320	miR-345	0,431	miR-345	0,242
4	cel-39	0,481	mir-1228	0,465	mir-193	0,263
5	mir-193	0,572	cel-39	0,485	miR-191	0,340
6					cell-39	0,347
Best combination	miR-520d, miR-1228	0.165	miR-345, miR-520d	0.223	miR-345, miR-520d	0.151

**Table 4:** Ranking of candidate reference miRNAs using BestKeeper algorithm.

Rank	Exosome		Plasma		Tissue	
	Gene name	Stability value	Gene name	Stability value	Gene name	Stability value
1	mir-1228	1.02	mir-520d	0.96	mir-345	0.82
2	mir-520d	1.25	mir-1228	1.22	mir-520d	0.93
3	mir-345	1.76	mir-193	1.53	mir-193	1.03
4	cell-39	1.87	mir-345	1.63	mir-1228	1.16
5	mir-193	2.39	cell-39	1.79	cell-39	1.49
6					mir-191	1.59

Despite the presence of modest inconsistencies due to differences in calculation methods between the two algorithms, the results obtained with BestKeeper were in accord with those obtained using NormFinder, thus confirming miR-520d at the top of the rank in all three matrices and miR-1228 and miR-345 as the second most stable miRNAs.

### Effect of suitable reference genes on relative expression of target miR-1290

**Figure 3:**
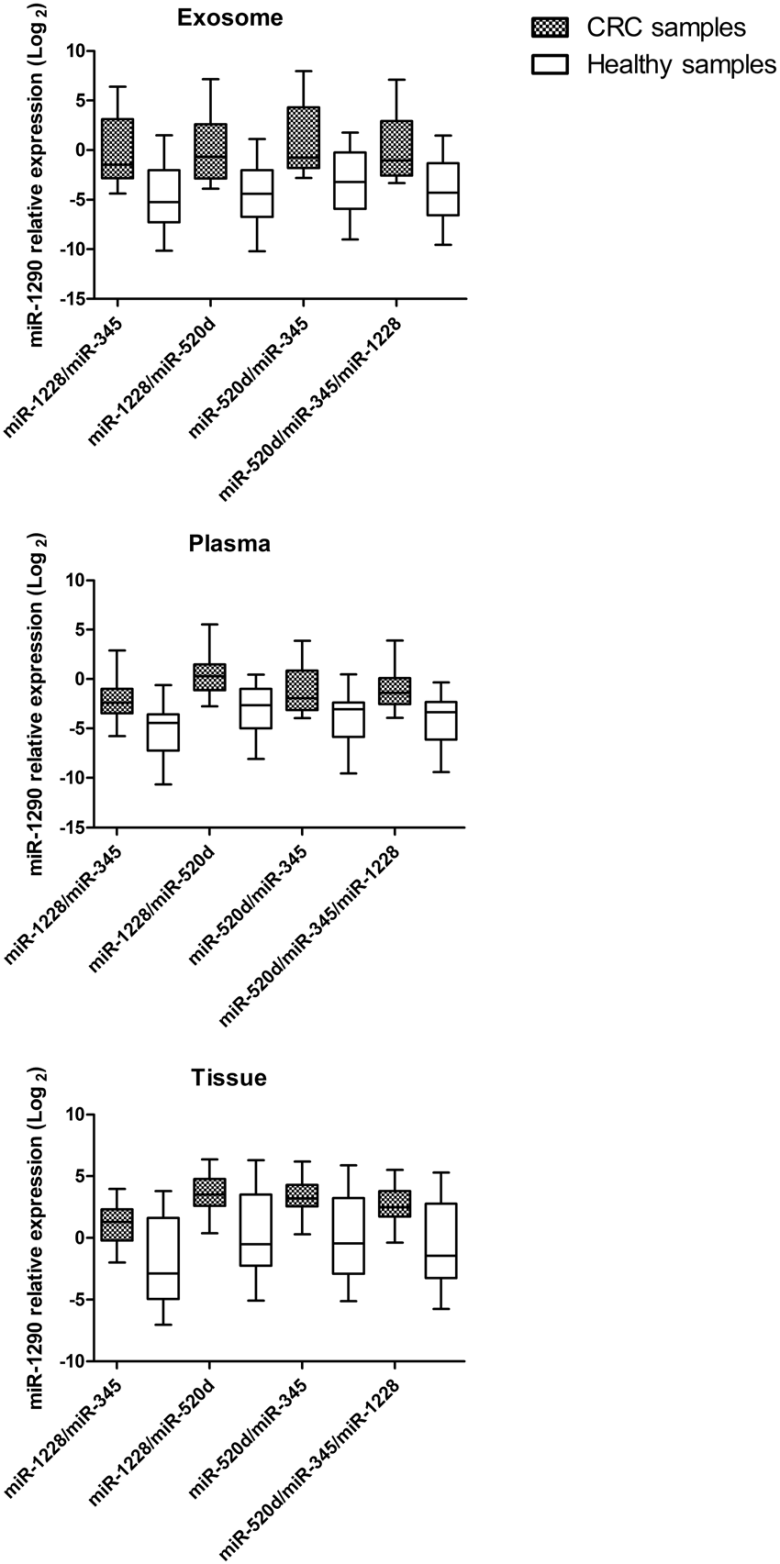
Effect of reference gene choice on relative expression of target miR-1290. By using different combinations of selected reference miRNAs the difference in miR-1290 expression between CRC and control samples always resulted significantly different (p < 0.005 for all).

The expression of miR-1290 in exosome, plasma and tissue of CRC patients and heathy controls was evaluated using single candidate reference gene as well as different combination of miR-1228, miR-345, miR-520d used for normalization. As shown in Fig. 3, all combinations allowed to identify a significant up-regulation of miR-1290 in exosome, plasma and tissue samples (all p < 0.005). The combination of miR-520d and miR-1228 led to the greatest difference in miR-1290 relative expression between CRC samples and controls in all the three matrices (p = 0.0002 for exosome, p = 0.0001 for plasma and 0.0051 for tissue). Conversely, no difference in miR-1290 expression could be detected when single, less stable reference genes were used for normalization (supplementary Figure 1).

## Discussion

Several lines of evidence suggest that the assessment of miRNAs not only hold great promise for the diagnosis, prognosis, prognostication and therapeutic follow-up of patients with CRC, but is can also be proposed as a potential tool for developing new cancer drugs^25^. However, appropriate use of miRNAs requires to clearly define the precise dysregulation of the different miRNAs in various disease states.

The normalization of miRNAs expression by using reference genes is an essential component of a reliable qPCR assay. This approach enables reducing the bias attributable to variations in extraction, reverse-transcription yield and amplification efficiency, so allowing the comparison of miRNA expression levels among different samples as well as the identification of longitudinal changes over time.

Although it is increasingly recognized that normalization of miRNA expression is a crucial aspect for obtaining reliable data, and that the use of inappropriate reference genes for normalization may generate unreliable or misleading results, putative housekeeping genes are still used as normalizers with no appropriate validation^26^. The current guidelines clearly recommend to identify stably expressed genes in each experimental system^27^, so generating a steady increase of studies aimed to dissect suitable normalizers in different pathological conditions and matrices, by using high throughput analyses. Although it is no longer accepted that certain reference genes are stable by convention, thanks to increasing understanding of miRNAs biology it is reasonable to assume that certain genes can be considered unstable a priori in a specific pathological condition. For example, owing to the well-established involvement of miR-16 in the pathogenesis of human cancer^28^, and due to its widely demonstrated down regulation in different malignancies^29–34^, it is now widely accepted that the conventional use of miR-16 as a normalizer should be abandoned in cancer research. A more rational approach for searching stable miRNAs within defined experimental protocol may start with candidate reference miRNAs already identified in the literature instead from using information obtained by means of high throughput techniques, as recently suggested by Schwarzenbach^13^.

One of the aims of this study was to perform a systematic review of the current scientific literature for identifying reliable evidence about potential reference miRNAs in CRC detected with high throughput technologies. Seven studies finally met our inclusion criteria. Due to heterogeneity of experimental design, sample matrices and population cohorts, a number of different candidates miRNAs have been identified. The only exception was miR-191, which was classified as the most stably expressed miRNAs by two independent research groups^18, 22^. The second part of our study encompassed the analysis of 12 miRNAs, which have been proposed as candidate reference miRNAs by the different studies. More specifically, we performed in silico analysis to test for their potential involvement in cancer biology. Among the number of bioinformatics tools available to search for miRNA-mRNA interaction^35^, we decided to perform a simple comparison between two databases. The former, miRTarBase, provides a manually based coxllection of current validated miRNA-target interactions (MTIs), whereas the latter, UniProTKB, contains a comprehensive list of known oncogenes and tumor suppressor genes. In order to exclude the largest number of potentially unstable miRNAs, matched data were analyzed by a conservative approach, so excluding from further analysis those miRNAs which had at least one oncogene or tumor suppressor gene among their validated targets. Seven miRNAs were so excluded (miR-16, miR-27a, let-7g, miR-25, miR-152, miR-132 and miR-103a). All these short RNA fragments target at least two onco/tumor suppressor genes and at least one gene involved in the pathogenesis of CRC (Additional file 2)^4, 36, 37^. Notably, the analysis on miR-16 revealed that, among the 52 putative targets, 11 were oncogenes or tumor suppressor genes (9 and 4 respectively). Four of these were found to have a definite role in the pathogenesis of CRC^4, 36, 37^. These are: *KRAS, CCND1, RAF* and *TP53*.

The miR-191, miR-193, miR-1228, miR-520d and miR-345 were instead miRNAs for which a significant associations with either onco- nor tumor-suppressor genes could not be proven. The performance of these candidate miRNAs, along with the spike in cel-39, was hence tested in the experimental part of our study. We performed, for the first time, the simultaneous evaluation of miRNAs expression levels and stability in CRC patients and healthy controls, using different biological matrices such as exosomes, plasma and CRC tissue samples.

The Cq values of *C. elegance* miR-39, which was used as a spike-in RNA control to define the quality control and confidence of qPCR performance, were highly reproducible among samples, displaying a median (standard deviation) SD of 2.1 across different biological matrices, so indicating that our data can be considered relatively robust.

According to the analysis of ∆Cq and the results obtained from NormFinder and Bestkeeper, miR-193 was found to be rather stable in plasma, and even more stable in tissue samples. Nevertheless, this miRNA displayed high ∆Cq and low stability values in exosomes, so that its use as reference miRNA may be discouraged in this matrix. Interestingly, we found that miR-191 was over-expressed in exosome and plasma sample of CRC patients compared to the exosomes and plasma of healthy controls. A broad ∆Cq was found for the analysis of miR-191 in both plasma samples and exosomes. The analysis of miR-191 Cq values by the two algorithms revealed low stability values in all the matrices. These results, rather consistent among tissue samples, plasma and exosomes, indicate that miR-191 may be implicated in the pathogenesis of CRC. Functional miRNA-cancer target interactions have not been earlier validated as with our in silico analysis, which failed to show any match with putative onco-targets in current databases. However, recent studies indicate that miR-191 may be strongly implicated in cancer biology^38^. According to our experimental data, miR-191 was found to be over expressed in more than 20 malignancies (including CRC), and this evidence is consistent with its classification as oncogenic miRNA. In CRC, anti-miR-191 attenuates invasiveness, suppresses proliferation and induces apoptosis by restoring the expression of tissue inhibitor of metalloprotease 3 (*TIMP3*), which is a potential target of this miRNA^39^.

Our combined analyses finally allowed to identify miR-520d, miR-345 and miR-1228 as the top three reference miRNAs in our study cohort. These miRNAs have been previously validated as suitable reference miRNAs in CRC tissue (miR-345) and plasma samples (mir-1228 and miR-520d)^8, 20, 21^. Therefore, their use in biological matrices different from those used in the primary studies need further evaluations having been tested here in a small sample set. However, the consistent stability displayed by these miRNAs across three different matrices prompt for their classification as “true reference miRNAs” in CRC, a conclusion that is also supported by their non-functional role in CRC as emerging from the current scientific literature.

miR-1228 was proven to be extensively involved in metabolism-related signal pathways and organ morphology. An unstable expression of this miRNA during development of the hematological system was also found by functional network analysis, so explaining its steady expression in blood^20^. miR-345 was found to play a major role in modulation of monocyte-related inflammatory response, through regulation of RelA transcription factor^40^. This miRNA is also involved in suppressing prostate cancer proliferation, invasion and migration, probably by downregulation of Smad1^41^. Human miR-520d is currently consider a minor miRNA, involved in HER2/neu receptor-related and osteoblast differentiation^42^. A recent study highlighted a key role of miR-520d in transformation of hepatoma cells into normal/benign phenotypes^43^.

By using different combination of these selected reference genes, a significant difference in miR-1290 expression was always detected in the three matrices. Contrarily, the results of target miR-1290 quantification were inconsistent when single, less stable miRNAs were used for normalization, thus highlighting the importance of selecting appropriated normalizers and confirming previous evidence that the use of more than one reference genes may enhance the accuracy of target miRNA quantitation^44, 45^.

Taken together, our results support the current opinion that the era of searching for universal reference miRNAs may be over^44, 45^. The cases of miR-16 and miR-191 presented here is a clear clue that the attitude to select a priori universal normalizers for miRNAs should be replaced in favor of adopting standardized methods for selecting case-specific normalizers. Although the application of high throughput screening is still considered the most effective benchmark, alternative and less expensive strategies exist^44, 45^. The approach used in our study has taken great benefit from the vast information gathered in over 20 years of investigating functional roles of miRNAs in both physiological and pathological states, as well as from advances in miRNAs expression studies and technologies. It consists of a series of sequential steps, as follows: (i) rigorous analysis of the literature for identifying candidate normalizers, (ii) analysis of the main interactions of miRNA-target through bioinformatics tools, (iii) elimination of miRNAs known to be involved in the pathogenesis of the study-specific disease, (iv) experimental verification of stability of candidate miRNAs within the specific study cohort and, (v) use of validated algorithms for final selection of the more suitable miRNAs for each experimental protocol.

Our approach for identification and verification of reference miRNAs in different biological matrices will hopefully represent a significant advancement to be used in future studies on CRC, but may also offer meaningful opportunities for other diseases and experimental conditions.

## Methods

### Systematic review

We carried out an electronic search for identifying studies which have assessed the stability of reference miRNAs in CRC. The preliminary search was performed in Medline (PubMed interface) from 2001 to March 7, 2016 without language restriction. The following keywords were used: “miRNA” AND “normaliz*” OR “stability” OR “control” OR “housekeeping” OR “reference” AND “colorectal” OR “colon” AND “cancer” OR “neoplasm” OR “malignancy”. We selected original articles of both observational studies and clinical trials when published in full text or full access to original data could be obtained. We hence included those studies in which the authors (i) selected the most stably expressed miRNAs from high-throughput technology-based miRNA expression profiling in a “discovery phase”(ii) assessed the stability of selected miRNAs by qPCR analysis in a “validation phase”. Two reviewers (ED and MM) independently evaluated titles and abstracts of the different publications to assess eligibility. A flow diagram of the study selection process is shown in Fig. 1.

Full data were extracted from all the publications meeting our inclusion criteria. The following information was also retrieved: first author’s last name, year of publication, country of origin; for both discovery and the validation studies: measurement method, analysis of data, source and number of case and control samples.

### *In silico* analysis

The target genes of candidate reference miRNAs selected from the literature were retrieved from miRTarBase^23^ (available at http://mirtarbase.mbc.nctu.edu.tw/), which includes the most updated and comprehensive information of experimentally validated miRNA-target interactions (last update, Sept. 15, 2015). The targets of the reference miRNAs were then compared with the list of human ‘oncogenes’ and ‘tumour suppressor genes’ retrieved from the UniProt Knowledgebase, a central hub for collection of functional information on proteins with accurate, consistent and extensive annotation^24^. Of the two UniProtKB sections, we selected that merging experimental results, computed features and scientific conclusions (UniProtKB/Swiss-Prot) with high quality manually-annotated and nonredundant records (last update June 21, 2016). The other, based on non-reviewed automatically annotated records (UniProtKB/TrEMBL) was not considered (Keyword: “Proto-oncogene [KW-0656]”; http://www.uniprot.org/uniprot/?query = KW-0656&sort = score), (Keyword: “Tumor suppressor [KW-0043]”; http://www.uniprot.org/uniprot/?query = KW-0043&sort = score). MiRNAs having no targets in both oncogenes and tumor suppressor genes were finally selected for the experimental verification study.

### Experimental study

#### Patients and specimens

The CRC specimens including tumor tissue, normal mucosa, plasma and exosome were obtained from 20 patients undergoing surgical resection at the University Hospital of Verona between 2010 and 2011 (mean age, 60 ± 13.5 years; 14 males and 6 females). Plasma and exosome were also obtained from 20 healthy volunteers enrolled from the staff of the local laboratory (mean age, 57.3 ± 9.2 years; 11 males and 9 females). Paired specimens of tumor and adjacent normal mucosa were obtained during surgical procedure, immediately frozen in liquid nitrogen and stored at −80 °C. According to the American Joint Committee on Cancer (AJCC) staging system^46^, 8 patients were at stage II, 8 patients at stage III, and 4 at stage IV. Only patients with primary colorectal adenocarcinomas untreated with neoadjuvant radio-chemotherapy were included in this study. Blood specimens were collected before intervention. Clinical information was accessed through hospital medical records. The study protocol was approved by the University Hospital of Verona Institutional Review Board. Informed consent was obtained from all patients prior to the collection of samples. Specimens and all experimental procedures were handled and carried out in accordance with the approved guidelines.

#### Extraction of exosomal, plasma and tissue RNA and conversion into cDNA

Isolation of total exosome and pretreatment of plasma and tissue specimens was performed according to standard procedures and as previously described^47–49^. RNA was extracted from plasma and exosome specimens with mirVana PARIS kit (Life Technologies, New York, USA) and Total *Exosome RNA and Protein Isolation* Kit (Life Technologies, New York, USA). Total RNA was isolated from normal mucosa and tumor tissues using a commercially available preparation (TriReagent; Molecular Research Center, Inc., Cincinnati, OH, USA). A detailed description of these procedures is available in the Supplementary methods.

After extraction, the RNA was quantified in triplicate on a NanoDrop ND-1000 Spectrophotometer (Thermo Scientific, Wilmington, Delaware, USA). The mean concentration of three measurements within the same RNA specimen was used to calculate the input total RNA for RT-qPCR. Reverse transcription was performed by the TaqMan Advanced miRNA cDNA synthesis Kit (Applied Biosystems, Life Technologies, Carlsbad, CA, USA). Briefly, 2 μl of total RNAs (corresponding to 10 ng of RNA), including miRNAs, were extended by a poly(A) tailing reaction using 0,06 U poly(A) polymerase enzyme and 1 mM of ATP. MiRNAs were then modified by lengthening the 5′ end by adaptor ligation. The miRNA with a poly(A) tail and adaptor was converted into cDNA through reverse transcription. Each reverse transcriptional reaction solution contained 1.2 μl of 25 mM dNTPs, 3 μl of 10X Reverse Transcriptase, 6 μl of 5x Reverse Transcription Buffer, 1.5 μl of 20 × Universal RT primer, 3.3 μl nuclease-free water. The reaction was carried out at 42 °C for 15 min and 85 °C for 5 min, using AB 2720 (Thermo Scientific, Wilmington, Delaware, USA). The samples of cDNA were stored at −20 °C for future usage.

#### Quantitative real-time PCR

Due to the low levels of some exosomal miRNAs, a preamplification step of cDNA was included in all quantifications. Five μl cDNA were preamplified in 25 μl of 2X miR-amp master mix, 2.5 μl of 20 × miR-amp primer mix and 17.5 μl nuclease-free water. PCR was run on AB 2720 (Thermo Scientific, Wilmington, Delaware, USA): 1 cycle at 95 °C for 5 min, 24 cycles at 95 °C for 3 s, 60 °C for 30 s and a terminal cycle at 99 °C for 5 min. The PCR products at the end of the run were diluted 1:7 in 0.1X TE buffer pH8.0 and stored at −20 ^◦^C. For quantitative real-time PCR, the miRNA-specific T*aqMan*™ *Advanced miRNA assays Kit* (Applied Biosystems Life Technologies, Carlsbad, CA, USA) for cell-39, miR-191, miR-193, miR-345, miR-520d miR-1228 and for the target oncogenic miR-1290 were used. Since probes for the small noncoding RNA RNU6 were not yet included in the Advanced kit, this small internal control was excluded from the analysis. In a 20 μl-reaction, 5 μl preamplified cDNA diluted was mixed with 10 μl 2X Fast Advanced Master mix, 1 μl 20 × miRNA specific TaqMan Advanced miRNA Assay and 4 μl nuclease-free water. Quantitative real-time PCR reaction was performed at 95 °C for 20 s and in 40 cycles at 95 °C for 3 s and 60 °C for 30 s, on a AB7500 Real Time System (Applied Biosystems Life Technologies, Carlsbad, CA, USA).

### Statistical analysis

The statistical analysis was performed using the Graph Pad Prism software package, version 5.0 (GraphPad software Inc., La Jolla,CA, USA). Distribution of continuous data was determined using the D’Agostino & Pearson omnibus test. The quantification cycle (Cq) value, which is inversely proportional to the target mRNA abundance, was used to estimate the level of gene expression. The mean of three repeated measures per sample was finally reported.

The expression levels of the candidate reference miRNA in patients and controls was assessed using box-plot analysis^50^. The Mann-Whitney test was used to assess the difference in relative expression levels of miRNAs between patients and controls. The expression variation range of Cq values (ΔCq) was calculated using the formula ΔCq = Cq_max_ − Cq_min_. Because the cDNA templates for the different samples were reverse transcribed from the same amount of total RNA, we used the ΔCq as the first parameter to assess expression stability of candidate reference miRNAs. A narrow range of Cq values indicated more stable expression. The expression stability was then evaluated by using two algorithms, i.e., NormFinder^51^ and BestKeeper^52^. Since these models assume that candidates should be stably expressed in different experimental groups, only miRNAs showing no statistically different expression between CRC and control samples were included in our analysis.

Briefly, the NormFinder uses an ANOVA-based model to separate the analysis of sample subgroups, estimates both the intra- and the intergroup expression variations, and finally calculates the stability value of a candidate gene. The program operates with a Microsoft Excel platform that automatically calculates the stability value for all candidate normalization genes containing any number of samples arranged in any number of groups. The most stable gene expression is indicated by the lowest average expression stability value. For NormFinder analyses, Cq values were transformed to relative quantities using the formula 2^−∆Cq^, where ∆Cq = Cq of the gene in selected sample - minimum Cq of corresponding gene in the experiment. The sample with minimum Cq or maximum expression was used as the calibrator with a set value of 1.

Bestkeeper (http://gene-quantification.com/bestkeeper.html) determines the geometric mean and standard deviation (SD) of Cq values of the candidate genes by pairwise correlation analyses. The lowest SD value indicates the most stable reference miRNA expression. Finally, the consensus rankings of the candidate reference miRNAs were determined according to the geometric rank means from the two analyses.

The relative expression of the target miR-1290 normalized to one or more reference candidates was also determined using the 2^−∆Cq^ method. The choice of this case study target miRNA was based on a recent finding according to which miR-1290 was found to be strongly up-regulated in plasma of patients with CRC^53^. The Mann-Whitney test was used to compare the expression of miR-1290 among the clinical groups.

## Electronic supplementary material

Figure S1

## References

[1] Cekaite L, Eide PW, Lind GE, Skotheim RI, Lothe RA (2016). MicroRNAs as growth regulators, their function and biomarker status in colorectal cancer. Oncotarget.

[2] Hollis M (2015). MicroRNAs potential utility in colon cancer: Early detection, prognosis, and chemosensitivity. World J Gastroenterol.

[3] Yiu AJ, Yiu CY (2016). Biomarkers in Colorectal Cancer. Anticancer Res.

[4] Thomas J, Ohtsuka M, Pichler M, Ling H (2015). MicroRNAs: Clinical Relevance in Colorectal Cancer. Int J Mol Sci.

[5] Weng W, Feng J, Qin H, Ma Y, Goel A (2015). An update on miRNAs as biological and clinical determinants in colorectal cancer: a bench-to-bedside approach. Future Oncol.

[6] Witwer KW (2015). Circulating microRNA biomarker studies: pitfalls and potential solutions. Clin Chem.

[7] Eriksen AH (2016). MicroRNA Expression Profiling to Identify and Validate Reference Genes for the Relative Quantification of microRNA in Rectal Cancer. PLoS One.

[8] Chang KH, Mestdagh P, Vandesompele J, Kerin MJ, Miller N (2010). MicroRNA expression profiling to identify and validate reference genes for relative quantification in colorectal cancer. BMC Cancer.

[9] Charkiewicz R (2016). Validation for histology-driven diagnosis in non-small cell lung cancer using hsa-miR-205 and hsa-miR-21 expression by two different normalization strategies. Int J Cancer.

[10] Pizzamiglio S (2014). A normalization strategy for the analysis of plasma microRNA qPCR data in colorectal cancer. Int J Cancer.

[11] Kang K, Peng X, Luo J, Gou D (2012). Identification of circulating miRNA biomarkers based on global quantitative real-time PCR profiling. J Anim Sci Biotechnol.

[12] Leitão MC (2014). Quantifying mRNA and microRNA with qPCR in cervical carcinogenesis: a validation of reference genes to ensure accurate data.. 2014 Nov 3. PLoS One.

[13] Schwarzenbach H, da Silva AM, Calin G, Pantel K (2015). Data Normalization Strategies for MicroRNA Quantification. Clin Chem.

[14] Nugent M, Miller N, Kerin MJ (2012). Circulating miR-34a levels are reduced in colorectal cancer. J Surg Oncol.

[15] Huang Z (2010). Plasma microRNAs are promising novel biomarkers for early detection of colorectal cancer. Int J Cancer.

[16] Ogata-Kawata H (2014). Circulating exosomal microRNAs as biomarkers of colon cancer. PLoS One.

[17] Xiang M (2014). U6 is not a suitable endogenous control for the quantification of circulating microRNAs. Biochem Biophys Res Commun.

[18] Zheng G (2013). Identification and validation of reference genes for qPCR detection of serum microRNAs in colorectal adenocarcinoma patients. PLoS One.

[19] Boisen MK (2015). MicroRNA Expression in Formalin-fixed Paraffin-embedded Cancer Tissue: Identifying Reference MicroRNAs and Variability. BMC Cancer.

[20] Hu J (2014). Human miR-1228 as a stable endogenous control for the quantification of circulating microRNAs in cancer patients. Int J Cancer.

[21] Rice J, Roberts H, Rai SN, Galandiuk S (2015). Housekeeping genes for studies of plasma microRNA: A need for more precise standardization. Surgery.

[22] Peltier HJ, Latham GJ (2008). Normalization of microRNA expression levels in quantitative RT-PCR assays: identification of suitable reference. RNA targets in normal and cancerous human solid tissues. RNA.

[23] Chou CH (2016). miRTarBase 2016: updates to the experimentally validated miRNA-target interactions database. Nucleic Acids Res.

[24] UniProt Consortium. UniProt: a hub for protein information. *Nucleic Acids Res* **43**, D204–D212 (2015). 10.1093/nar/gku989 PMC4384041 25348405

[25] Beermann J, Piccoli MT, Viereck J, Thum T (2016). Non-coding RNAs in Development and Disease: Background, Mechanisms, and Therapeutic Approaches.. 2016. Physiol Rev.

[26] Gutierrez, L. *et al*. The lack of a systematic validation of reference genes: a serious pitfall undervalued in reverse transcription-polymerase chain reaction (RT-PCR) analysis in plants. *Plant Biotechnol J* **6** (2008). 10.1111/j.1467-7652.2008.00346.x 18433420

[27] Bustin SA (2009). The MIQE guidelines: minimum information for publication of quantitative real-time PCR experiments. Clin Chem.

[28] Aqeilan RI, Calin GA, Croce CM (2010). miR-15a and miR-16-1 in cancer: discovery, function and future perspectives. Cell Death Differ.

[29] Delfino KR, Rodriguez-Zas SL (2013). Transcription factormicroRNA- target gene networks associated with ovarian cancer survival and recurrence. PLoS One.

[30] Ell B (2013). Tumor-induced osteoclast miRNA changes as regulators and biomarkers of osteolytic bone metastasis. Cancer Cell.

[31] Hu Z (2012). Serum microRNA profiling and breast cancer risk: The use of mir-484/191 as endogenous controls. Carcinogenesis.

[32] Liu J (2012). Combination of plasma microRNAs with serum CA19–9 for early detection of pancreatic cancer. Int J Cancer.

[33] Xiao G (2014). Aberrant expression of microRNA-15a and microRNA-16 synergistically associates with tumor progression and prognosis in patients with colorectal cancer. Gastroenterol Res Pract.

[34] Zuo Z (2011). Circulating microRNAs let-7a and miR-16 predict progression-free survival and overall survival in patients with myelodysplastic syndrome. Blood.

[35] Akhtar MM, Micolucci L, Islam MS, Olivieri F, Procopio AD (2016). Bioinformatic tools for microRNA dissection. Nucleic Acids Res.

[36] Colussi D, Brandi G, Bazzoli F, Ricciardiello LColussiD (2013). Molecular pathways involved in colorectal cancer: implications for disease behavior and prevention. Int J Mol Sci.

[37] Valeri N, Croce CM, Fabbri M (2009). Pathogenetic and clinical relevance of microRNAs in colorectal cancer. Cancer Genomics Proteomics.

[38] Nagpal N, Kulshreshtha R (2014). miR-191: an emerging player in disease biology. Front Genet.

[39] Qin S (2014). MicroRNA-191 correlates with poor prognosis of colorectal carcinoma and plays multiple roles by targeting tissue inhibitor of metalloprotease 3. Neoplasma.

[40] Dang, T. M. *et al*. MicroRNA expression profiling of human blood monocyte subsets highlights functional differences. Immunology 145 (2015). 10.1111/imm.12456 PMC4479539 25707426

[41] Chen QG (2016). MiR-345 suppresses proliferation, migration and invasion by targeting Smad1 in human prostate cancer. J Cancer Res Clin Oncol.

[42] Lowery AJ (2009). MicroRNA signatures predict oestrogen receptor, progesterone receptor and HER2/neu receptor status in breast cancer. Breast Cancer Res.

[43] Tsuno S, Wang X, Shomori K, Hasegawa J, Miura N (2014). Hsa-miR-520d induces hepatoma cells to form normal liver tissues via a stemness-mediated process. Sci Rep.

[44] Marabita F (2016). Normalization of circulating microRNA expression data obtained by quantitative real-time RT-PCR. Brief Bioinform.

[45] Schlosser K, McIntyre LA, White RJ, Stewart DJ (2015). Customized Internal Reference Controls for Improved Assessment of Circulating MicroRNAs in Disease. PLoS One.

[46] Edge SB, Compton CC (2010). The American Joint Committee on Cancer: the 7th edition of the AJCC cancer staging manual and the future of TNM. Ann Surg Oncol.

[47] Danese E (2013). Epigenetic alteration: new insights moving from tissue to plasma - the example of PCDH10 promoter methylation in colorectal cancer. 2013 Aug 6. Br J Cancer.

[48] Danese E (2015). Comparison of genetic and epigenetic alterations of primary tumors and matched plasma samples in patients with colorectal cancer. PLoS One.

[49] Danese E (2010). Real-time polymerase chain reaction quantification of free DNA in serum of patients with polyps and colorectal cancers. Clin Chem Lab Med.

[50] Royeen CB (1986). The boxplot: a screening test for research data. Am J Occup Ther.

[51] Andersen CL, Jensen JL, Orntoft TF (2004). Normalization of real-time quantitative reverse transcription-PCR data: a model-based variance estimation approach to identify genes suited for normalization, applied to bladder and colon cancer data sets. Cancer Res.

[52] Pfaffl MW, Tichopad A, Prgomet C, Neuvians TP (2004). Determination of stable housekeeping genes, differentially regulated target genes and sample integrity: BestKeeper–Excel-based tool using pair-wise correlations. Biotechnol. lett.

[53] Imaoka H (2016). Circulating microRNA-1290 as a novel diagnostic and prognostic biomarker in human colorectal cancer. Ann. Oncol..

