# Supplementary material for: Reference miRNAs for colorectal cancer: analysis and verification of current data
Source: Sci Rep. 2017 Aug 21;7:8413. doi: 10.1038/s41598-017-08784-3 (PMC5567181; doi:10.1038/s41598-017-08784-3)
Supplement: Supplementary file 1 — Figure S1 [file 41598_2017_8784_MOESM1_ESM.doc]

**Reference miRNAs for colorectal cancer: analysis and verification of current data**

Danese E*^1^, Minicozzi AM^2#^, Benati M^1#^, Paviati E^1^, Lima-Oliveira G^1^, Gusella M^3^, Pasini F^3^, Salvagno GL^1^, Montagnana M^1^, Lippi G^1^.

1. Clinical Biochemistry section, Department of Neurosciences, Biomedicine and Movement Sciences, University of Verona, Verona, Italy
2. Colorectal & Peritoneal Oncology Centre, The Christie NHS Foundation Trust, Manchester Manchester M20 4BX, United Kingdom.
3. Laboratory of Pharmacology and Molecular Biology, Oncology Department, Rovigo General Hospital, 45027 Trecenta, Italy.
4. Department of Medical Oncology, Rovigo Hospital, 45100 Rovigo, Italy.

#Equally contributed to this work

**Figure S1**: Effect of reference gene choice on relative expression of target miR-1290 in CRC (n=20) and healthy samples (n=20). miR-1290 resulted up-regulated in CRC when the follow reference miRNAs were used for normalization: cell-39 (p=0.022)and miR-1228 for exosome (p=0.015); miR-1228 (p=0.033) and miR-520d (p=0.034) for plasma; miR-191 (p=0.044), miR-193 (p=0.002) and miR-520d (p=0.001) for tissue.
